# Current trends in textile wastewater treatment—bibliometric review

**DOI:** 10.1007/s11356-024-32454-3

**Published:** 2024-02-21

**Authors:** Mohammad Tajul Islam, Md. Abdullah Al Mamun, Abul Fazal Mohammad Fahad Halim, Roberta Peila, Diego Omar Sanchez Ramirez

**Affiliations:** 1 Department of Textile Engineering, Ahsanullah University of Science and Technology, Dhaka, Bangladesh; 2 Department of Corporate Leadership and Marketing, Szechenyi Istvan University, Gyor, Hungary; 3 Centre for Water, Energy and Waste, Murdoch University, Perth, Australia; 4 CNR-STIIMA (National Research Council of Italy-Institute of Intelligent Industrial Technologies and Systems for Advanced Manufacturing), Biella, Italy

**Keywords:** Oxidation, Advanced oxidation process, Membranes, Ultra/nano-filtration, Adsorbent nanomaterials, Electrolysis

## Abstract

A bibliometric study using 1992 to 2021 database of the Science Citation Index Expanded was carried out to identify which are the current trends in textile wastewater treatment research. The study aimed to analyze the performance of scholarly scientific communications in terms of yearly publications/citations, total citations, scientific journals, and their categories in the Web of Sciences, top institutions/countries and research trends. The annual publication of scientific articles fluctuated in the first ten years, with a steady decrease for the last twenty years. An analysis of the most common terms used in the authors’ keywords, publications’ titles, and KeyWords Plus was carried out to predict future trends and current research priorities. Adsorbent nanomaterials would be the future of wastewater treatment for decoloration of the residual dyes in the wastewater. Membranes and electrolysis are important to demineralize textile effluent for reusing wastewater. Modern filtration techniques such as ultrafiltration and nanofiltration are advanced membrane filtration applications.

## Introduction

Due to the significant amount of water used in various wet processing procedures, the textile sector was ranked as the 3rd largest source of water consumption in 2020 (European Parliament 2020). According to information reported by European Commission in the Best Available Techniques Reference document for the Textiles Industry, the amount of waste water discharged across 95 emission plants in the European Union is ranged between 0.01 and 696 m^3^ t^-1^ of textiles treated, average value 79.6 (Roth et al. 2023). It has been also shown that the dyeing and finishing of textile products require a substantial amount of water (Islam 2016). Because of that, the textile sector is among the three greatest global water demand sectors after food and recreation/culture (European Environment Agency), and almost all of its discharged wastewater is extremely contaminated. Wastewater discharged from the textile industries may contain many chemical components such as acids/alkalis, bleaches/colorants, ethylenediaminetetraacetic acid (EDTA), surfactants (wetting agents and soaps), and heavy metals ions (i.e., Hg, As, and Pb) (Paul et al. 2012). The World Bank estimates that between 17 and 20% of industrial wastewater might be produced during the dyeing and finishing of clothes (Ding et al. 2010). Diverse compounds in wastewater cause limited light transmittance, which has a negative impact on plant photosynthesis and, in turn, results in aquatic creatures consuming less oxygen. The textile industry currently uses aromatic and heterocyclic dyes. These intricate and stable dye structures pose more severe wastewater degradation issues than any other type of complex lattice (Ding et al. 2010). A great effort has been put into sustainable chemicals (Halim et al. 2021), environmentally friendly dyeing (Islam 2016; Islam et al. 2020), printing (Islam et al. 2016), and textile finishing (Islam and Asaduzzaman 2019; Hoque et al. 2021). Despite all the efforts, the water that is constantly contaminated by the textile industry needs to be decolorized before being released into the environment. In this case, significant ecological concerns arise, for example, the mineralization of dyes, the effect of organic chemicals, and their harmfulness for the environment. Therefore, it is important for the environment that proper textile wastewater management is understood and developed.

**Fig. 1:**
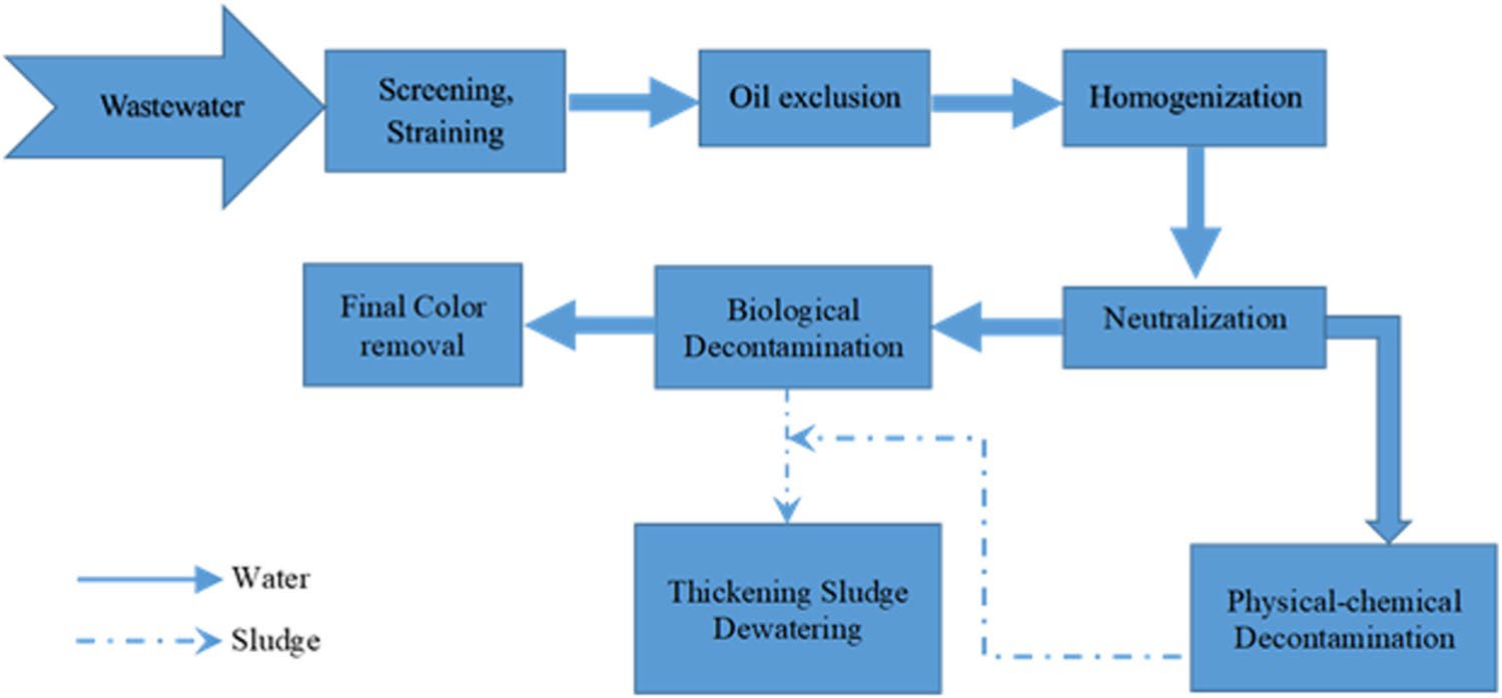
A standard outline of wastewater treatment in a typical textile industry

Fats, oils, pigments, and other chemicals used in numerous industrial steps must be taken out of the wastewater (Fig. 1). Considering the fact that not all factories employ the same production technique, the amount and type of water emitted have to be taken into account to determine the cleaning procedure. Furthermore, not all manufacturers utilize the same chemicals; in particular, businesses located in countries with high environmental standards try to maintain the same water quality throughout the whole production process. As a result, the approach behind water treatment could be different.

**Fig. 2:**
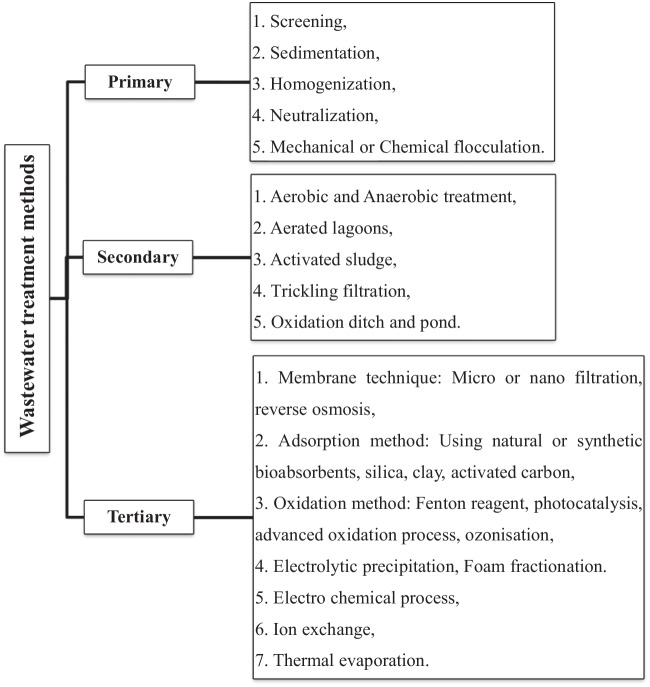
Wastewater treatment methods

Different technologies and pieces of equipment have been created and put to the test in laboratory, pilot, or industrial scale. These technologies are linked with the general idea of wastewater purification, and it is necessary to take into account the particular circumstances of textile factories (Park and Shore 1984; Athanasopoulos 1990). It is advised to separate various wastewater types into the following categories in the first step: concentrated fluids, moderately polluted wastes, and slight to zero contaminated wastes. Either at the workplace or at sewage treatment facilities, wastewater can be treated. The benefit of purifying wastewater in textile factories is that water may be reused in most cases. The wastewater treatment methods can be summarized as shown in Fig. 2.

Bibliometrics is a visualization study to map published information on a research field. The research trend in specialized areas of study has been tracked successfully by bibliometric analysis such as pigment coloration research (Islam et al. 2022), wound dressing (Ho et al. 2022b), and bacterial nanocellulose (Ho et al. 2022a). The research methodology used by bibliometrics is based on statistics and quantitative analysis. This method is widely employed in information sciences and library. This research method helps unveil and identify the distribution trends of published articles in any database for a chosen field, institution, country and research topic. A database is required to perform a bibliometric analysis. Scopus of Elsevier and Clarivate Analytics’s the Science Citation Index Expanded (SCIE) are two significant datasets used by the researchers. The Web of Science (WoS) Core Collection of Clarivate Analytics has six indexing databases of which SCIE is for scientific disciplines. Here, SCIE was employed to extract the published materials for profiling the scientific achievements in textile wastewater research worldwide.

The studies on textile wastewater over the last three decades was examined to understand this field’s global research situation better. This bibliometric analysis also provides a basis for developing medium and long-term strategies for textile wastewater. Thus, the study systematically covered statistical descriptions of published scientific materials and their performance in various scales and indicators such as major journals, journal categories, impact factors of journals, yearly publication rate, and geographical location of researchers and research organizations. Moreover, the identification of research trends and focal points was accomplished by examining the titles of papers, as well as analyzing the KeyWords Plus and keywords provided by the authors.

## Data and bibliometric methods

In this work, the data were acquired from Clarivate Analytics via the SCIE of WoS platform, with the most recent update conducted on November 22, 2021. On June 28, 2022, Journal Citation Reports released their 2021 journal impact factors (JIF). As per the JIF’s definition, it is recommended to consider papers published during 2021, specifically after June 30, 2022, from the SCIE database. Despite being primarily designed for academics to explore and locate the literature, the data from SCIE is not readily available in a format suitable for bibliometric analyses (Ho 2018). Because of this, data treatment is always needed for bibliometric investigations after collecting data directly from SCIE. A noticeable disparity has recently been observed when employing the title/abstract of article and its author keywords as filters in traditional bibliometric studies (Ho 2020). Through the extraction of supplementary words from the titles of papers cited within bibliographies/footnotes found in the Clarivate Analytics database, KeyWords Plus can effectively augment and enrich the existing title-based and author-provided keywords (Garfield 1990). Indeed, it has been noted that the records found by using only KeyWords Plus were irrelevant during the search topic. (Fu and Ho 2015). Search keywords “treatment,” “treatments,” “textile,” “textiles,” “wastewater,” “wastewaters,” “waste water,” “waste waters,” “effluent,” “effluents”, “effluent treatment plant,” and “effluent treatment plants” were searched in the titles, the author keywords, and abstract fields in the SCIE. 5275 documents are produced between 1991 and 2021. On their “first page,” only 128 documents (2.4% of the 5275 documents) lack searchable keywords. These 5147 documents were regarded as publications on textile wastewater treatment. In order to analyze all 5147 records, a spreadsheet containing the raw data was created using Microsoft Excel 2016. (Li and Ho 2008). Furthermore, each JIF2021 was determined by utilizing the 2021 Journal Citation Reports.

Due to the fact that the term “corresponding author” is listed as a reprint author in SCIE, it was utilized in the context of this analysis. If more than one author contributed to a given publication, the last corresponding author was designated to be “corresponding author,” and their affiliation served as “corresponding author” information. When the corresponding authorship was not explicitly stated, the sole author was regarded the first and “corresponding author” of the work. Likewise, in cases where a publication had a single institution associated with it, that particular institution was identified as the institution of the first and “corresponding author” (Ho 2014a).

Affiliations coming from Scotland, England, Wales, and Northern Ireland were taken to be works from the United Kingdom (UK) to have proper analytical results (Chiu and Ho 2005). The citations of a publication were examined using four citation metrics, as follows:

- *C*_*0*_ is the total count of citations from the WoS Core Collection during the year of publication (Ho 2014b).
- *C*_*year*_ is the total count of citations received in a specific year from the WoS Core Collection. For instance, *C*_*2021*_ indicates the citations received during the year 2021 (Ho 2012).
- *TC*_*year*_ is the cumulative total of citations from the WoS Core Collection, starting from the publication year and extending up to the most recent year (Wang and Ho 2011). In this work, the most recent year is 2021, denoted as *TC*_*2021*_.
- *CPP*_*year*_ is the mean number of citations per publication, calculated as *TC*_*2021*_ divided by the total count of publications (TP) (Ho 2012).

## Results and discussion

### Document type and language of publication

The amount of citations for each publication has been linked to the type of document, according to certain reports (Hsieh et al. 2004). The utilization of the *CPP*_*year*_ citation indicator in 2015 led to an enhancement in the measurements of citations per publication, offering more precise and accurate values (Ho and Ho 2015). The mean number of authors per publication (*MAP)* has recently come up when discussing various document forms. Table 1 displays the attributes of 10 different document types, including 4480 articles (or 87% of the total 5147 documents), and 4.4 writers on average per publication (*MAP*). The papers about textile wastewater treatment with the most writers (Mattioli et al. 2005) had 19 authors. The highest *CPP*_*2021*_ was for the document type of reviews, at 130.8. Five widely cited evaluations with a *TC*_*2021*_ of 1000 or more are responsible for that which were by Verma et al. (2012), Rafatullah et al. (2010), Lee et al. (2016), Robinson et al. (Robinson et al. 2001), and Pearce et al. (Pearce et al. 2003) with a *TC*_*2021*_ of 1087, 1930, 1141, 3564, and 1120, respectively. A total of 291 reviews, mostly in Water Science And Technology, were widely published in 159 publications (13 reviews; 4.4% of 291 reviews). The book reviews’ CPP_2021_ was 2.5 times higher than those of the articles. With a TC_2021_ of 138, one classic review with a TC_2021_ of above 100 was published by (Abdel-Hamid et al. 2013). In addition, textile wastewater treatment research constituted seven out of the top 10 most frequently cited publications. The documents of WoS Core Collection could be divided up into multiple categories. For instance, 145 documents were categorized as proceedings, papers, and article document categories. As a result, the overall percentage is greater than 100% (Usman and Ho 2020).

**Table 1:** Total publication, authors, and citation distribution for the document type

Document type	TP	TP-AI	%	TA	MAP	*TC*_*2021*_	*CPP*_2021_
Article	4480	4480	87.04	19,811	4.4	127,116	28.4
Book chapter	2	2	0.04	3	1.5	4	2
Book review	2	2	0.04	5	2.5	142	71
Correction	19	19	0.37	31	1.6	0	0
Data paper	3	3	0.06	13	4.3	15	5
Editorial material	9	9	0.17	29	3.2	77	8.6
Meeting abstract	14	14	0.27	67	4.8	6	0.4
Note	3	3	0.06	8	2.7	87	29
Proceedings paper	324	324	6.29	1180	3.6	1078	3.3
Review	291	291	5.65	1139	3.9	38,051	130.8

*TP-AI* total count of publications using author information available in SCIE. *%* percentage of 5147 articles, *TA* the total count of authors, *MAP* mean number of authors per publication

From all document categories, only 5147 articles were chosen for additional examination because they include a comprehensive research presentation. The introduction, procedures, results, analyses, and conclusions are all included in those articles. The language of publication stands as a pivotal factor in bibliometric studies, particularly in the context of big data analysis (Wang and Ho 2011). There were 5147 articles totaling 15 languages. A substantial majority of articles (97.5%) were composed in the English language. A smaller proportion of articles were written in other languages including Portuguese (54; 1%), Spanish (14; 0.27%), Turkish (12; 0.23%), and Croatian following distantly (11; 0.21%). French (10 articles), Polish (8 articles), German (4 articles), Chinese (4 articles), Russian (3 articles), Korean (2 articles), Czech (2 articles), Slovene (2 articles), Japanese (2 articles), and one in a bilingual newspaper were written in Serbo-Croatian. Articles in English language had a CPP_2021_ of 33, which was significantly greater than non-English-language articles' CPP_2021_, which was just 7.5. Articles in English-language had an MAP of 4.4, which was greater than those in non-English-language MAP of 3.5.

### Characteristics of publication output

**Fig. 3:**
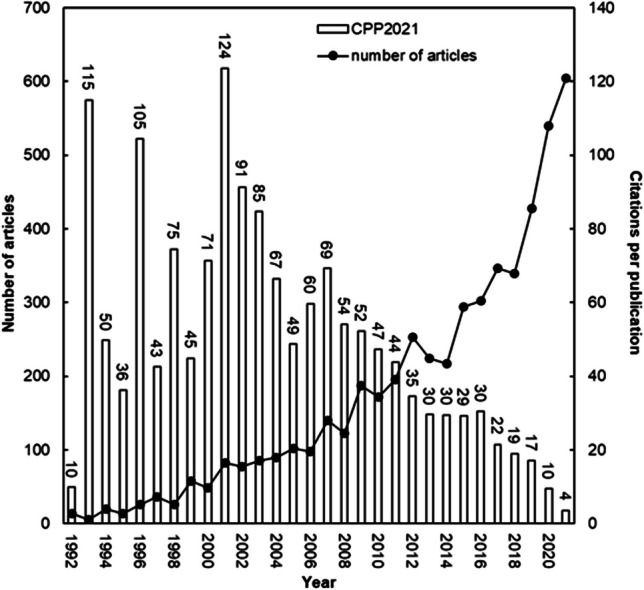
TP and CPP_year_ values over time

It is possible to assess the influence of publications on research disciplines using the *CPP*_*year*_ value (Ho 2013). In fact, the distribution of TP and *CPP*_*year*_ is illustrated on a yearly basis in Fig. 3. The year 2001 had the highest *CPP*_*year*_ of 124 with 82 articles. A total of 14 papers, or 17% of the 82 articles published in 2001, received 100 or more highly citations, with one classic piece receiving 1000 or more. (Houas et al. 2001). *CPP*_*2021*_ has been declining since 2001, but TP has been experiencing a faster growth rate, reaching a count of 600 in the year 2021. This observation implies that the field of textile wastewater treatment research has garnered significant attention from researchers. As a result, the value of *CPP*_*year*_ is bound to grow over time as additional articles are released annually.

### WoS categories and journals

**Fig. 4:**
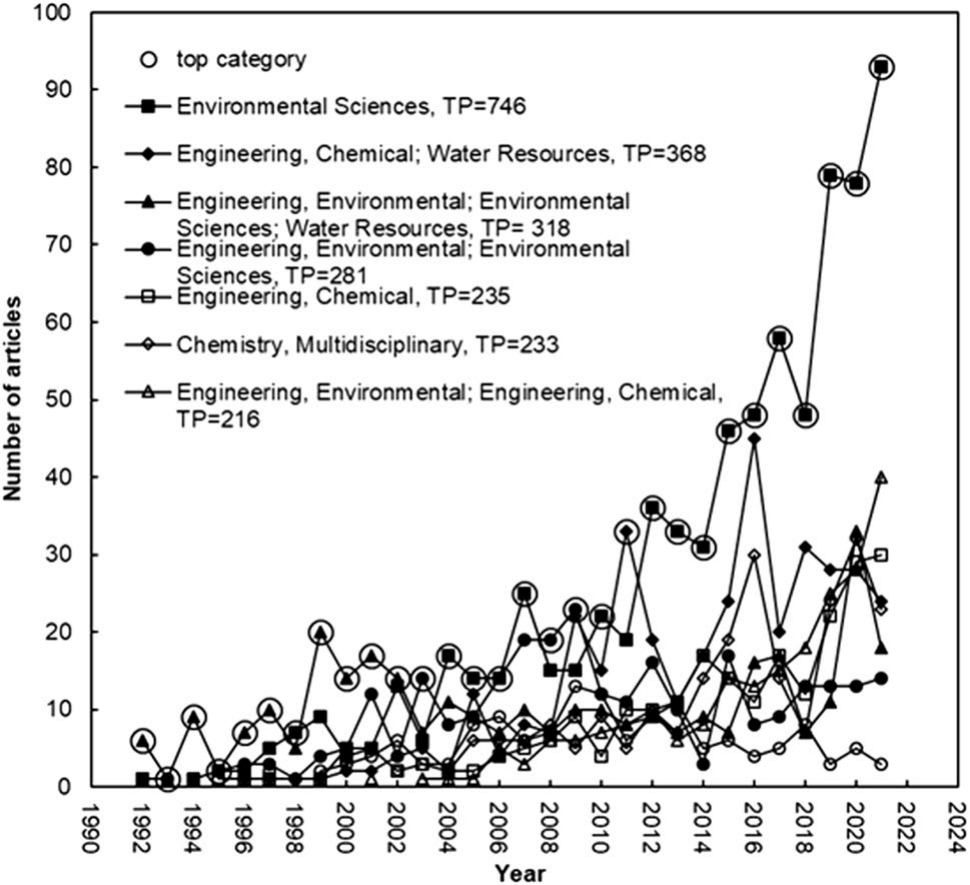
TP over time for the top nine WoS categories (TP > 200)

Publications (5147) on textile wastewater treatment were published in 1036 journals across a wide range of disciplines and 186 WoS categories in SCIE. Table 2 displays the top ten WoS categories. Environmental Sciences was the most popular category on WoS with 746 articles (14.4% of 5147 entries), then Engineering, Chemical, and Water Resources (368; 7.1%). The publications on textile wastewater treatment with the highest *CPP*_*2021*_ = 59.9 among this top was published in the Biotechnology & Applied Microbiology category. Physical Chemistry, a subcategory of chemistry, published 80 publications (ranked 13th; 1.5% of 5147 entries), with a high *CPP*_*2021*_ of 39.8. Engineering, Environmental and Engineering, Chemical categories had a *MAP* of 4.6, whereas Materials Science, Textiles had the lowest average with a value of 3.3. Journals listed within the WoS index can be categorized into two or more distinct categories; for instance, the *Chemical Engineering Journal* was categorized under both Chemical and Environmental Engineering. Therefore, the total of the percentages can exceed 100%. The expansion of the leading seven categories, each comprising 200 or more publications, is portrayed in Fig. 4. Environmental Sciences has been publishing articles on textile wastewater remediation since 1991. Since 1992, articles pertaining to textile wastewater treatment have been published in the Engineering, Chemical category. Water resources, chemistry, and Biotechnology & Applied Microbiology are three multidisciplinary disciplines that have recently become most popular and have been rated 2nd, 6th, and 8th by 2021, respectively. Engineering, Environmental; Environmental Sciences obtained a total of 281 articles published (ranked 4th), however just 14 were published in 2021 (ranked 11th in 2020).

**Table 2:** The top 10 WoS categories for textile wastewater treatment research

WoS category	*TP* (%)	*TC*_2021_	*CPP*_2021_	*TA*	*MAP*
Environmental Sciences	746 (14.1)	24,998	33.5	3349	4.5
Engineering, Chemical; Water Resources	368 (7)	8801	23.9	1515	4.1
Engineering, Environmental; Environmental Sciences; Water Resources	318 (6)	12,926	40.6	1252	3.9
Engineering, Environmental; Environmental Sciences	281 (5.3)	15,440	54.9	1158	4.1
Engineering, Chemical	235 (4.5)	7703	32.8	1057	4.5
Chemistry, Multidisciplinary	233 (4.4)	3419	14.7	982	4.2
Engineering, Environmental; Engineering, Chemical	216 (4.1)	8937	41.4	989	4.6
Biotechnology & Applied Microbiology	143 (2.7)	8559	59.9	580	4.1
Materials Science, Textiles	116 (2.2)	1011	8.7	386	3.3
Green & Sustainable Science & Technology; Engineering, Environmental; Environmental Sciences	109 (2.1)	3449	31.6	498	4.6

Desalination and Water Treatment (JIF2021 = 1.254), Water Science and Technology (JIF2021 = 2.430), Journal of Hazardous Materials (JIF2021 = 14.224), Environmental Science and Pollution Research (JIF2021 = 5.190), and Chemosphere (JIF2021 = 8.943) were among the 5 most productive journals and these had 100 publications or more (2.2%).

### Publication performance by countries/institutions

The analysis did not include the 12 articles (0.22% of 5147 papers) because the authors’affiliation information was missing from SCIE. Out of the 5147 textile wastewater treatment publications produced by 97 nations, 4070 (or 79%) were produced in only one nation while 1077 (or 21%) were the result of international collaborations including 67 nations. Table 3 reports the 10 most productive nations. This top included five Asian countries, two European countries, one each from South and North America, and one from across continents. Under of this top, it can also be found Australia (66 articles, at position 23), and Egypt (128 articles, at position 13) like the most producing country in Africa.

**Table 3:** Top 10 countries publishing research on textile wastewater treatment

Country	TP	*TPR* (%)	*TP CPP*_2021_	*IPR* (%)	*CPR* (%)	*FPR* (%)	*FP CPP*_2021_	*RPR* (%)	*RP CPP*_2021_	*SPR* (%)
India	896	1 (17.4)	32.9	1 (17.9)	2 (16.8)	1 (16.5)	29.9	1 (15.8)	30.1	2 (7.8)
China	602	2 (11.7)	22.8	2 (10.6)	1 (17.4)	2 (10.4)	22.3	2 (10.3)	21.8	5 (4.1)
Brazil	480	3 (9.3)	28.2	3 (9.1)	4 (11.1)	3 (8.7)	28.4	3 (8.4)	29	33 (0.5)
Turkey	415	4 (8.1)	29.4	4 (9)	18 (4.8)	4 (7.7)	28.9	4 (7.8)	29.7	1 (18.1)
USA	235	5 (4.6)	38.3	10 (2.4)	3 (14.1)	10 (2.5)	39.9	10 (2.6)	40.9	3 (5.2)
Pakistan	235	6 (4.6)	20.7	6 (3.3)	6 (10.4)	5 (3.9)	19.3	5 (3.9)	18.9	14 (1.6)
Iran	206	7 (4)	28.9	5 (4.2)	19 (4.8)	6 (3.7)	28.7	6 (3.6)	28.8	6 (3.6)
Spain	199	8 (3.9)	44.6	7 (3.3)	10 (6.7)	8 (3.1)	44.8	8 (3)	40	12 (2.1)
Malaysia	191	9 (3.7)	47.1	8 (3.2)	15 (6.1)	7 (3.2)	49.3	7 (3.3)	47.4	24 (0.5)
Italy	178	10 (3.5)	39.1	9 (2.8)	12 (6.4)	9 (2.8)	33.8	9 (2.7)	33.3	15 (1.6)

*TPR* (%) rank based on the total count of articles and the corresponding %, *IPR* (%) rank of single-country articles and their % among all single-country articles, *CPR* (%) rank of internationally collaborative articles and their % among all globally collaborative articles, *FPR* (%) rank of first-author articles and their % among all first-author articles, *RPR* (%) rank of corresponding author articles and their % among all corresponding author articles, *SPR* (%) rank of single-author articles and their % among all single-author articles

For the assessment of publication performance, six indicators were used: TP, articles with international collaboration (CP), articles from one country (IP), corresponding author articles (RP), articles with first authors (FP), and articles with one author (SP), as well as their CPP_2021_ (Hsuly and Ho 2014). India did better in the four indices, TP = 896 articles (17.4% of 5147 articles), IP = 731 articles (17.9% of 4070 articles from a single nation), FP = 847 articles (16.7% of 5055 first-author articles), and RP = 814 articles (16% of 5056 corresponding-author articles). One publication indication showed China to be in the lead, CP = 171 articles (17.3% of 985 internationally collaborative articles). In the remaining publication indication, Turkey achieved the highest score with SP = 35 articles (18.1% of 193 single-author articles). The greatest *CPP*_*2021*_ of TP, FP, and RP was found in textile wastewater treatment products from Malaysia, followed always by Spain, Italy, and United States of America (USA). Nevertheless, it is important to highlight that two of the top 10 articles on textile wastewater treatment were written by Malaysian authors (Rafatullah et al. 2010; Lee et al. 2016).

**Fig. 5:**
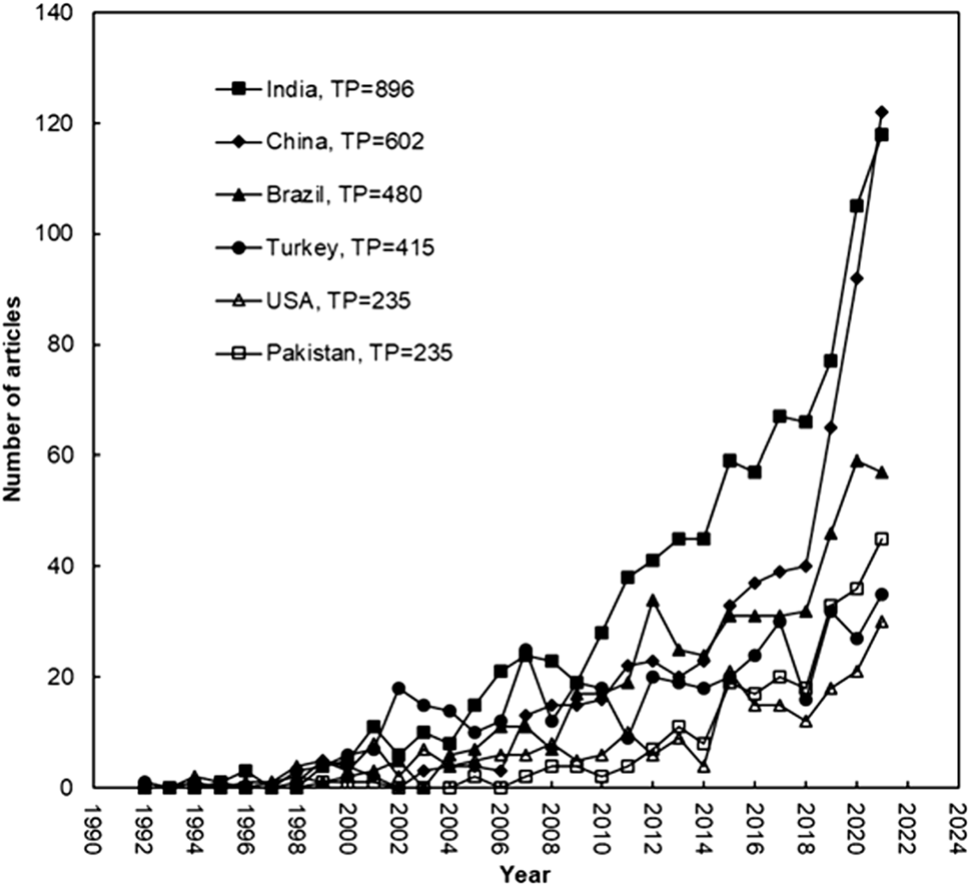
TP over time for the top six countries (*TP* > 200)

Figure 5 compares the six main countries with 200 or more publications in terms of their level of development. Before 2012, each nation had an annual publication count of less than 40 papers, with India contributing to the majority of these publications. Afterwards, it was seen an exponential growth of articles per year until reached 122 in 2021 for India and China. For the same year, the latter dominated the field of textile wastewater treatment research. Even though Pakistan published its first article in 1999, in the recent years, it have significantly increased its publications in this topic, which has helped this country to be ranked 6th in 2021 with 45 articles.

Out of a total of 5147 papers, 2321 (or 45%) were produced by a single institution, while 2734 (or 55%) were the outcome of collaborative efforts among institutions. Table 4 showcases the attributes of the ten most productive universities by six metrics indexes. With a TP of 124 articles (2.4% of 5147 articles), an IP of 72 articles (3.1% of 2321 single-institution articles), a CP of 52 articles (1.9% of 2734 collaborative-institution articles), an FP of 95 articles (1.8% of 5147 first author articles), and an RP with 92 articles, the Indian Institute of Technology has secured the first position in five different publication indicators. The Istanbul Technical University in Turkey took first place in one publication metric with an SP of 7 articles (3.6% of 193 single-institution articles).

**Table 4:** Top 10 productive institutions

Institute	TP	*TPR* (%)	*IPR* (%)	*CPR* (%)	*FPR* (%)	*RPR* (%)	*SPR* (%)	*CPP*_2021_
Indian Institute of Technology, India	124	1 (2.4)	1 (3)	1 (2)	1 (1.8)	1 (1.8)	2 (2.5)	47.4
Istanbul Technical University, Turkey	82	2 (1.5)	2 (1.7)	8 (1.3)	2 (1.2)	2 (1.2)	1 (4.5)	22.8
University of Agriculture Faisalabad, Pakistan	71	3 (1.4)	13 (0.7)	2 (2)	7 (0.8)	6 (0.7)	N/A	26.3
Universiti Teknologi Malaysia, Malaysia	66	4 (1.3)	3 (1.6)	12 (1.1)	4 (1)	3 (1)	N/A	24
National Institute of Technology, India	63	5 (1.2)	6 (1.2)	9 (1.3)	3 (1)	4 (0.9)	N/A	30.8
Donghua University, China	57	6 (1.1)	22 (0.5)	4 (1.6)	8 (0.8)	10 (0.7)	N/A	17.8
Anna University, India	55	7 (1.1)	4 (1.3)	16 (0.9)	5 (0.9)	5 (0.9)	N/A	24.4
Universidade Federal de Santa Catarina (UFSC), Brazil	55	8 (1)	9 (0.9)	10 (1.1)	9 (0.7)	11 (0.6)	N/A	34.9
Chinese Academy of Sciences, China	52	9 (1)	190 (0.1)	3 (1.8)	22 (0.5)	22 (0.4)	N/A	33.1
Lodz University of Technology, Poland	52	10 (1)	39 (0.3)	5 (1.6)	12 (0.6)	13 (0.6)	8 (1.3)	26.8

*N/A* not available

Poland’s Lodz University of Technology was one of the top three institutions to produce single-author articles (ranked 8th). The Lodz University of Technology scored the fifth-highest CPP_2021_ of 26.8 compared to the top 10 most prestigious institutions, and it was closely followed by the University of Agriculture Faisalabad in Pakistan. Chinese and Turkish institutions, in contrast, have lower CPP_2021_ values. The presence of multiple branches of the Indian Institute of Technology across numerous cities led to a bias. The publications of the institute with branches were regarded as belonging to a single institution; otherwise, various rankings would have been obtained.

### The most impact articles and the most frequently cited articles in 2021

Works that receive high references might not necessarily have a substantial impact/visibility after their publication (Ho and Kahn 2014). Readers can find further information regarding the impact of a highly referred work today by looking at the number of citations obtained in the most recent year, for this study was 2021 (C_2021_) (Ho 2012). In comparison to the ranking produced by the C_2021_ sorting, a different ranking was produced when 5147 textile wastewater treatment products were sorted using TC_2021_. Out of a total of 5147 publications, 1488 (28.9%) did not obtain citations during 2021 (C_2021_ = 0), and 639 (12.4%) got zero citations at all since their publication up to the end of 2021 (TC_2021_ = 0). Likewise, among the best 100 TC_2021_ papers, 52% of those were also at the top 100 C_2021_ publications. By using the title, abstract, and author keywords a total of 5147 textile wastewater treatment publications were obtained. It is worth remembering that the title of an article provides adequate information about the subjects of a paper (Wang et al. 2010). Additionally, the authors provide keywords to give more details about the main area of research in their publications. Nevertheless, the use of search terms in their abstracts might not directly address the topic of the search.

For example, two classic articles titled “Mutagenic and carcinogenic potential of a textile azo dye processing plant effluent that impacts a drinking water source” (Ohe et al. 2004) and “Mutagenic and carcinogenic potential of a textile azo dye processing plant effluent that impacts a drinking water source” (de Lima et al. 2007), and a highly cited article entitled “Chemistry, biochemistry, and safety of acrylamide. A review” (Friedman 2003) contained the search terms in their abstract only. However, these publications have nothing to do with the study of textile wastewater treatment.

**Fig. 6:**
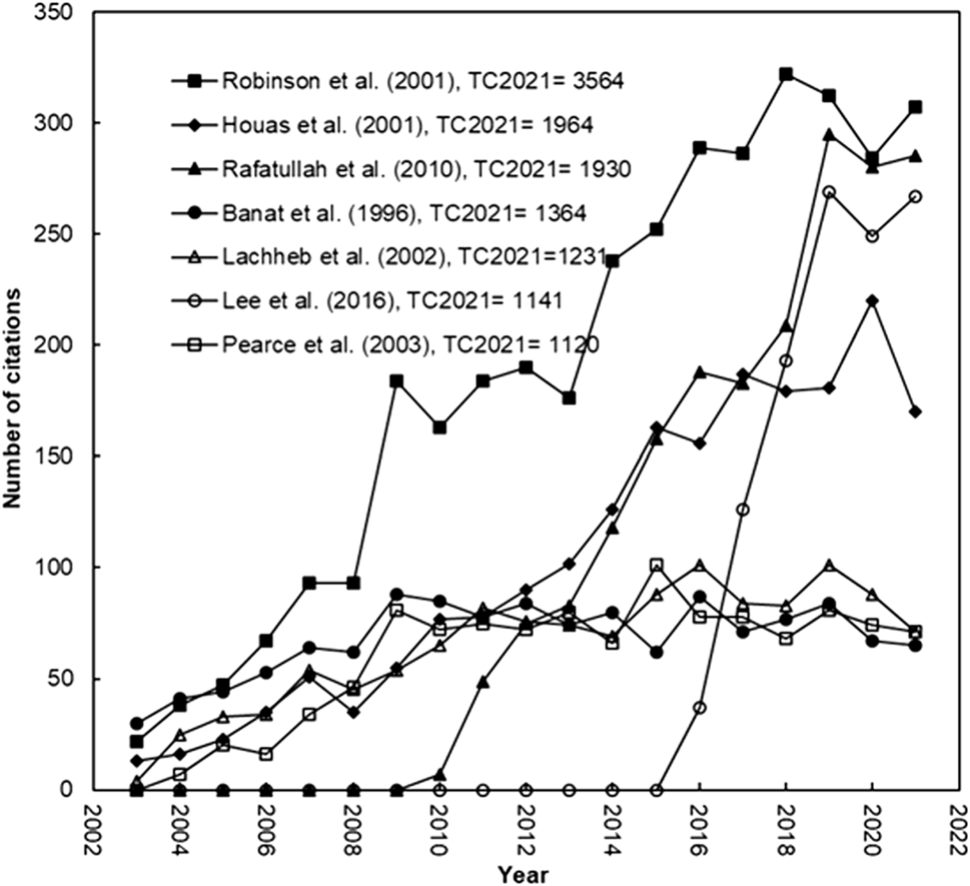
Citations over time for the seven most frequently cited articles, which include search keywords in their titles or author keywords

In all the titles, abstracts, and author keywords of the best 20 papers on *TC*_*2021*_, 18 of them contained search terms. Some of these works were done by Houas et al. (2001) ranked 2nd with *TC*_2021_ of 1964, Lachheb et al. (2002) ranked 5th with *TC*_2021_ of 1231, Duran et al. (2007) ranked 19th with *TC*_2021_ of 610, and Ajmal et al. (2014) ranked 20th with *TC*_2021_ of 584. The seven most cited publications, featuring search terms in their titles or author keywords, are showed in Fig. 6 along with their citation histories. The publication carried out by Rafatullah et al. (2010) ranked 3rd on *TC*_2021_ with 1930 but 2nd on *C*_*2021*_ with 285, showing a sharp increase in citations since 2010. Similarly, an article by Lee et al. (2016) ranked 6th on *TC*_2021_ with 1141 but ranked 4th on *C*_2021_ with 267 received a sharp increase in citations since 2013. The article entitled “Remediation of dyes in textile effluent: a critical review on current treatment technologies with a proposed alternative” by Robinson et al. (2001) from the University of Ulster of Coleraine in UK maintained a strong citation increase trend after its publication year. The ten articles with the greatest numbers of citations and search terms in the title or author keywords are listed in Table 5. These publications were mainly written by the UK, India, and Belgium. France, Malaysia, Tunisia, Italy, Taiwan, South Korea, Australia, Singapore, and Thailand also contributed an item in this list.

**Table 5:** The ten articles that are most frequently cited, and which include search keywords in their titles or author keywords

*R* (*TC*_2021_)	*R* (*C*_2021_)	Title	Country	Reference
1 (3564)	1 (307)	“Remediation of dyes in textile effluent: a critical review on current treatment technologies with a proposed alternative”	UK	(Robinson et al. 2001)
2 (1964)	7 (170)	“Photocatalytic degradation pathway of methylene blue in water”	France	(Houas et al. 2001)
3 (1930)	2 (285)	“Adsorption of methylene blue on low-cost adsorbents: A review”	Malaysia	(Rafatullah et al. 2010)
5 (1231)	35 (71)	“Photocatalytic degradation of various types of dyes (Alizarin S, Crocein Orange G, Methyl Red, Congo Red, Methylene Blue) in water by UV-irradiated titania”	Tunisia	(Lachheb et al. 2002)
7 (1120)	34 (71)	“The removal of colour from textile wastewater using whole bacterial cells: a review”	UK	(Pearce et al. 2003)
8 (1087)	6 (181)	“A review on chemical coagulation/flocculation technologies for removal of colour from textile wastewaters”	India	(Verma et al. 2012)
10 (856)	133 (33)	“Treatment and reuse of wastewater from the textile wet-processing industry: Review of emerging technologies”	Belgium, Italy	(Vandevivere et al. 1998)
12 (828)	12 (120)	“Bacterial decolorization and degradation of azo dyes: A review”	Taiwan, India, South Korea	(Saratale et al. 2011)
13 (813)	57 (52)	“Tailored titanium dioxide photocatalysts for the degradation of organic dyes in wastewater treatment: A review”	Australia, Singapore	(Han et al. 2009)
14 (760)	252 (22)	“White-rot fungi and their enzymes for the treatment of industrial dye effluents”	Belgium	(Wesenberg et al. 2003)

*R*: ranking in 5147 textile wastewater treatment articles

### Research trends

The distribution of terms in the abstract, article title, KeyWords Plus, and author keywords over time might provide useful information for identifying the main goals of the research and revealing how those goals have changed over time (Wang and Ho 2016). For the entire study length and the 30-year study period, words from article titles, author keywords, abstracts, and KeyWords Plus were analyzed and ranked in accordance. Removal, degradation, and decolorization were the most often used terms outside of search keywords.

**Fig. 7:**
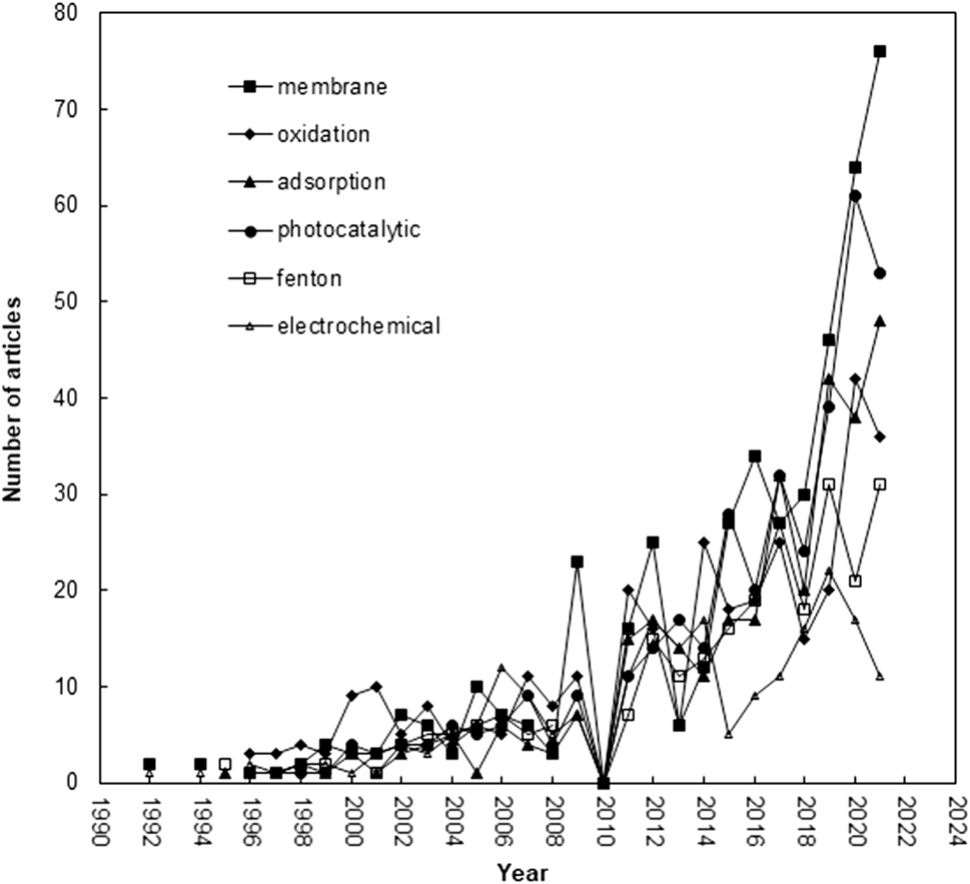
Main research topics in textile wastewater treatment

The following were the six potential research areas for textile wastewater treatment research: “oxidation,” “Advanced Oxidation Process,” “membrane,” “ultra- and nanofiltration,” “adsorbent nanomaterials,” and “electrolysis” are shown in Fig. 7. Each word cluster is made up of a number of supporting terms that were found through word analysis.

### Oxidation

Ozone is a very unstable molecule that decomposes in molecular oxygen in standard conditions. It was discovered that ozone decomposition in water causes the formation of highly oxidative hydroxy radicals that can interact with and disrupt many organic pollutants (Staehelin and Hoigne 1985). For this reason, ozone has been used in many applications in the past decades. Particularly in water disinfection to eliminate bacteria, water odor, and color. Many advantages arise in using ozone as an oxidizing agent, primarily because, generally, the reactions involved do not produce harmful compounds, so ozonation was considered a promising technology for contaminant degradation of wastewater. Several studies examined different oxidation methods for local wastewater treatment, including membranes, activated carbon (Joss et al. 2008), and hydrogen peroxide coupled with an iron tetramido-macrocyclic ligand (Jans et al. 2021). According to these works, ozonation turned out to be the most appropriate technology for wastewater treatment in several countries due to the broad range of micropollutants that can be degraded. Recent literature, however, raised some restrictions on the usage of ozonation treatment. Sometimes organic pollutants are not entirely removed or may generate harmful intermediates, such as R-COH and R-COOH, as well as disinfection by-products (Lim et al. 2022). Moreover, the poor ozone water solubility may involve bad process performance and elevated OPerational EXpenditure (Yang et al. 2021). To overcome these limitations, combining other methodologies with ozonation is under investigation. Wang and Chen described the recent advances and perspective of catalytic ozonation utilizing both homogeneous and heterogeneous catalysts (Wang and Chen 2020). The addition of a catalyst can improve the process performance and facilitate ozone decomposition, forming more active free radicals for the breakdown of toxic organic compounds.

Inhomogeneous catalytic ozonation, metal ions are generally employed as catalysts. The metal ions can react with ozone molecules enhancing their decomposition to generate hydroxyl radicals. During O_3_ decomposition, hydroperoxy radicals, HO_2_^− •^, form as well and allow the regeneration of the reduced metal ions. At the opposite, metal ions may combine with organic matter in a complex formation. These complexes are subsequently oxidized by ozone and other oxidizing species formed during the process (Psaltou et al. 2021).

Different metal oxides are employed in heterogeneous catalytic ozonation, such as manganese oxides, aluminum, iron-based compounds like oxides and oxyhydroxide, and activated carbon. The catalyst provides active sites to adsorb and catalyze reactions. Three different pathways are possible: the catalytic surface adsorb and decompose the ozone molecules to form free radicals which react with the toxic organic compounds in the solution; the catalyst can adsorb the toxic organic compounds that subsequently react with ozone molecules; the catalyst surface adsorb both toxic organic and ozone molecules and the reaction between them takes place on its surface (Wang and Chen 2020; Issaka et al. 2022; Yang et al. 2022).

Compared to homogeneous catalytic ozonation, the heterogeneous catalytic one has a higher catalytic activity based on the type of catalysts employed; moreover their separation from the liquid phase is simplified as they can be reused and do not cause secondary environment pollution (Liu et al. 2016). Van et al. used a heterogeneous catalyst derived from iron slag waste from metallurgical industry to degrade the azo-reactive dye red 24 (RR24) by ozonation from aqueous solutions. In this work, it was reported that under 11 water solution pH, the combination of ozone and iron slag, containing FeO, ZnO, and SiO_2_, showed a synergistic effect on RR24 dye and had a maximum efficiency for an iron slag concentration of 1 g L^−1^. The decolorization of RR24 achieved almost 100% (Van et al. 2019). Hu et al. employed the sol–gel and impregnation method to prepare the catalyst carbon aerogel-supported copper oxide. They evaluated its catalytic efficiency in the ozonation treatment of prepared textile wastewater for reactive black 5 (RB5) synthetic dye degradation. The chemical oxygen demand (COD) removal was 46% after 60 min by ozonation catalytic reaction in a semi-continuous reactor (Hu et al. 2016). A magnetic oxidized graphitic carbon nitride (g-C_3_N_4_)/Al_2_O_3_ nanocatalyst was prepared by Faghihinezhad et al. The COD elimination rate was four times higher than simple ozonation, and the biodegradability index of the real textile wastewater reached the textile discharge standards (Faghihinezhad et al. 2022).

Gao et al. studied the ozonation process to enhance ozone’s mass transfer. They modified the inner structure of a rotating bar reactor accordingly to different configurations of sieve plates and spoiler columns. An optimal internal structure of sieves with opening sizes of 0.5 and 1 mm exhibited a greater volumetric mass transfer coefficient and a greater decolorization rate for the reactive black 5 (RB5) synthetic dye (Gao et al. 2022a).

### Advanced oxidation process (AOP)

Among AOP, photocatalysis is one of the most effective technique in textile wastewater treatment from textile dye facilities and other manufacturing companies. AOP is an oxidation process involving forming a proper amount of hydroxyl radicals. These radicals have an extensive oxidation power and can promptly degrade organic and some inorganic pollutants of wastewater (Deng and Zhao 2015; Helmy et al. 2017). In photocatalysis, a semiconductor material is irradiated by a light source, like visible light, that excites the electrons of the valence band to the conduction band, creating a gap between e^−^/h^+^. That originates highly reactive species from the reaction with O_2_ and H_2_O, like superoxide radical anions and hydroxyl radicals. This process allows the oxidation and disruption of organic and inorganic pollutants (Rueda-Marquez et al. 2020). A review from Subhiksha et al. (2022) describes using bismuth-based photocatalysts (BBP) for wastewater treatment. They report the performances of different bismuth compounds, including bismuth oxide, sulfide, tungstate, vanadate, and molybdate in contaminant degradation. They all show a high oxidative capacity toward different types of pollutants. However, some limitations in their use are related to their low visible light absorption, slow migration of charge carriers, and charge recombination. To overcome these restraints, research is focused on BBP modification. Examples are reported for metal and non-metal ions doping as well as heterojunction by introducing a different semiconductor to improve the charge separation/transfer mechanism and enhance the BBP photocatalytic activity. For example, the system WO_3_/BiVO_4_ was more effective than WO_3_ and BiVO_4_ alone (Chen et al. 2013). BBP demonstrated an important antimicrobial activity and a reduction of Cr-containing pollutants.

Qui et al. reported a 90% inactivation of the gram-negative *E. coli* under visible light for Bi_2_O_3_ (Rajkumar and Kim 2006). A significant *E. coli* inactivation was found by Karbasi et al. as well (Karbasi et al. 2020). They studied the photocatalytic inactivation of bacteria using flower-shaped Bi_2_WO_6_ under visible light. In addition, researchers have shown the degradation of Cr^+^ containing pollutants. Gao et al. studied the BiVO_4_-Ni/AgVO_3_ heterostructure photocatalytic activity. It achieved a 99% of Cr^+^ reduction within 80 min. (Gao et al. 2022b) Mao et al. synthesized the eterojunction structure Zn_0.78_Cd_0.22_S/Bi_2_MoO_6_ employing ultrasounds that were able to degrade by photocatalysis the Cr (VI) ions (Mao et al. 2022).

Nanostructured materials are other highly efficient photocatalysts in degrading wastewater pollutants, particularly harmful synthetic dyes. Metal oxide nanoparticles are widely explored in photocatalysis due to their high surface area, good absorption of contaminants, and fast kinetic. Due to the high band-gap of metals, if irradiated by a light source, the h^+^/e^−^ hole is formed. That allows the reaction between the formed hole and the species adsorbed on the surface of the catalysts. Vinay et al. describe the ZnO nanoparticle synthesis from the natural source Areca nut from the *Areca catechu* plant. Areca nut extract was reacted with zinc nitrate hexahydrate in an aqueous solution to form ZnO nanoparticles, eventually separated from the colloidal solution. The photocatalytic activity of the synthesized ZnO nanoparticles was assessed for Rhodamine-B, Methylene Blue, and Nigrosine dyes under direct sunlight. The reactions were analyzed using UV–Vis spectroscopy. Rhodamine-B showed a 95% degradation after adsorption on ZnO nanoparticles and 120 min sunlight irradiation; Methylene Blue after 90 min and Nigrosine after 90 min (Raghavendra et al. 2022). TiO_2_ nanoparticles are other nanomaterials employed mainly in many research areas and photocatalysis. Chakraborty et al. realized 3D nanoflowers structures of TiO_2_ through hydrothermal synthesis (Chakraborty et al. 2022). Thanks to the 3D structure, this type of TiO_2_ possesses better physicochemical properties, a higher surface area, and adsorption sites than other nanostructures. Moreover, by studying the effect of concentration and solvent of solvent, temperature, pH of the reaction solution, reaction time, and titanium precursor, it was possible to reach a 99% organic pollutants degradation.

Many factors influence the photocatalytic mechanism of nanoparticles and must be considered to attain maximum efficiency. Among them, nanoparticles' size, surface area, and shape are critical factors in the photocatalytic mechanism since they are correlated to the number of active sites in the adsorption of contaminants (Xu et al. 2019; Dave and Jagtap 2022).

### Membranes

Nowadays, the textiles industries cause the major release of dyes into the environment, followed by paper/pulp, tannery, paint, and dye manufacturing (Samsami et al. 2020). A conventional technology used for dye removal is membrane filtration: microfiltration (MF), ultrafiltration (UF), nanofiltration (NF), and reverse osmosis (RO). By and large, MF and UF are considered pre-treatments to remove particles and macromolecules before NF/RO so that those can concentrate and retain ions, dyes, and small organic compounds (Allègre et al. 2006; Samsami et al. 2020). In the literature, it has been reported the treatment and reuse of textile effluents at pilot-scale and industrial scale using pre-treatments (MF or UF) and NF/RO to reach industrial and environmental requirements—dye and salt recovery over 90% for RO (Marcucci et al. 2001; Allègre et al. 2006; Abid et al. 2012). From an economic standpoint, NF offers more benefits because it requires a smaller operating pressure than RO. Under the same operating conditions, NF can reduce electricity consumption by about 50% and decrease fouling formation compared with RO (Liu et al. 2011; Abid et al. 2012).

In wastewater treatments, it is also possible to find forward osmosis (FO), whose driving force is the osmotic pressure gradient, through a semi-permeable membrane with a mean pore size of about 0.25 nm. This technology has been used in combination with coagulation/flocculation (CF) or membrane distillation (MD) to concentrate textile wastewater and diminish liquid discharges in the textile industry (Han et al. 2016; Li et al. 2020). FO has been showed to be a process with an excellent rejection toward dyes/salts (about 90%), reversible fouling, and lower energy consumption (Han et al. 2016; Li et al. 2020). In particular, MD is a thermally driven process that has reported its feasibility in textile wastewater treatment showing antifouling properties (An et al. 2016; Fortunato et al. 2021).

A membrane bioreactor (MBR) is another membrane separation process in textile wastewater treatment that uses MF/UF membranes and activated sludge units. In MBR, it is possible to grow microorganisms capable of degrading dyes into harmless compounds (Brik et al. 2006). Although MBR can achieve dye removal above 87% for textile wastewater treatment, this technique may require low sludge ages and NF membranes as a post-treatment to match superior removal values (Brik et al. 2006).

It is also possible to include inorganic materials (i.e., nanosheets and micro/nanoparticles) into polymeric membranes to overcome physical, chemical and thermal limitations of membranes in some environments during a purification process—these membranes are known as mixed matrix membranes (MMMs) (Basile et al. 2011; Zare and Kargari 2018). In the literature, UF membranes have been combined with adsorbents to produce hybrid membrane adsorption systems. In this way, dyes are not only separated by hollow fiber UF membrane (pore size between 100 and 10 nm) but also adsorbed by mesoporous MCM-41 (mean diameter particle 1230 nm, average pore diameter 2.5 nm, and pore wall thickness 1.37 nm) increasing dyes removal (Alardhi et al. 2020). For an actual textile wastewater, these membranes showed a dye removal above 97% (Alardhi et al. 2020). In another study, MMC-41 was embedded in polyethersulfone membranes for NFs applications and the amount of these mesoporous inside the membrane was optimized to tackle energy consumption during filtration by increasing the permeability to its maximum value (Kadhim et al. 2021). Polyethersulfone membranes with 0.8%wt. of MCM-41 and a feed dye concentration of 10 ppm reported the maximum permeability value at 64 and 63 L m^−2^ h^−1^ bar^−1^ for 938 Da—acid black 210 and 1018 Da—rose bengal, respectively (Kadhim et al. 2021).

2-D materials can also be employed in membranes to treat textile wastewater. For example, the use of MoS_2_ nanosheets and oxidized multi-walled carbon nanotubes blended on polyethersulfone membrane (mean pore size 21 nm) demonstrated an increasing permeability (64 L m^−2^ h^−1^ bar^−1^), a better antifouling property, a high removal for dye (98% for 1136 Da − reactive red 195 and 94% for 627 Da—reactive blue 19), and protein (99% 66.43 kDa—bovine serum albumin) (Arefi-Oskoui et al. 2022). Indeed, interlayered nanosheets of MXene (Ti_3_C_2_T_x_) were also used in polyamide FO membrane showing a variation for the mean pore size (and water permeability) from 370 nm (4406 L m^−2^ h^−1^ bar^−1^) to 230 nm (3829 L m^−2^ h^−1^ bar^−1^). Likewise, interlayered MXene-FO membranes increased their permeability, salt rejection, and potential application in organic solvent recovery (Wu et al. 2020). Finally, graphene oxide nanosheets (monolayer structure of graphite) have also been incorporated into polyethersulfone to increase the hydrophilicity of membranes in NF applications, which in turn can improve its permeability value during dye separation (Al-Sultan et al. 2022). After optimizing graphene oxide content, polyethersulfone/graphene oxide membrane (mean pore size on the surface between 14.58 and 15.58 nm) showed the best permeability value at 123 L m^−2^ h^−1^ bar^−1^ for 1%wt. of graphene oxide with a feed dye concentration of 10 ppm using 1018 Da—rose bengal (Al-Sultan et al. 2022).

On the other hand, membranes can be functionalized with photocatalytic properties using nanoparticles (NPs) to separate and degrade dyes from wastewater simultaneously. In the literature, the development of asymmetric membranes (pore size 200 nm) made of cellulose acetate/polyurethane with ZnO-NPs has also been reported (Rajeswari et al. 2017). These membranes demonstrated a degradation efficiency of over 90% (illumination time 40 min, pH 7, and initial concentration 50 mg L^−1^) for 681 Da—reactive red 11 and 1850 Da—reactive orange 84 (Rajeswari et al. 2017).

In the literature was also reported a case where hydroxyapatite-based bio-ceramic hollow fiber membrane has been used to separate dyes and heavy metals from textile effluents (Hubadillah et al. 2020).This ceramic membrane (mean pore size 13 nm) exhibited a dye and heavy metal ion separation (Cu, Fe, Zn, Cr, and Cd) of 99.9 and 100%, respectively (Hubadillah et al. 2020).

### UF and NF membranes

UF and NF membranes can be sorted out by their molecular weight cut-off (MWCO): UF—5000–10,000 Da, tight-UF (T-UF)—1000–5000 Da, loose-NF (L-NF)—500–1000 Da, and NF—200–1000 Da (Ye et al. 2020b, a). Generally, NF membranes have excellent dye removal (80–100%) and salt rejection (70–90%) under different operating conditions (Avlonitis et al. 2008). But even so, polyamide thin-film composite NF membrane can be modified using diethanolamine (DEA) so that these membranes (MWCO 290 Da) could have better permeability, antifouling properties, and excellent dye removal (99% methyl blue—800 Da, 99% congo red—697 Da, 98% sunset yellow—452 Da, and 80% neutral red—289 Da) (Liu et al. 2017). Analogously, UF membranes can reach dye removal over 90% considering membrane MWCO, dye MW, and the aggregation of dyes. For example, UF membranes (mean pore size 1.5 nm) with MWCO between 17,530 Da and 6050 Da have shown a dye removal over 92% (1373 Da—direct red 80, 840 Da—reactive blue 2, 814 Da—direct red 23, and 618 Da—reactive orange 16) (Jiang et al. 2018).

However, it is necessary to point out that NF membranes (pore size 2 nm) commonly reach a better dye removal in comparison with UF (pore size 20–50 nm) 40% and 90%, respectively (Fersi et al. 2005). Furthermore, contrary to the previous case, a UF membrane (pore size 1–100 nm) of 1000 Da can also remove 100% everzol blue (629 Da) and everzol red (788 Da), while a NF membrane (pore size 1 nm) of 200–300 Da could only remove the same dyes over 90% (Aouni et al. 2012). But even so, NF membranes are more appropriate than UF membranes to separate dyes from textile wastewater because the former has better salt retention than the latter (Fersi et al. 2005; Aouni et al. 2012).

CF is a simple and efficient pre-treatment that uses coagulants and flocculants to reduce dye content up to 90% (Khouni et al. 2011). In combination with UF or NF membranes, it is possible to achieve dyes reduction of 74% (several dyes of textile plant effluent) and 99% (Black Novacron R and Blue Bezaktiv S-GLD 150), respectively (Khouni et al. 2011).

It is essential to highlight that NF membranes are usually hydrophobic and negatively charged at operating conditions (Xu et al. 2016). Moreover, charged NF membranes could have antifouling properties and better permeation than those without charges (Zhu et al. 2014; Xu et al. 2016). As a result, NF membranes can be functionalized with other polymers or nanomaterials with positive/negative charges and more hydrophilic properties. In the literature, NF membranes with positive charges were obtained using catechol and branched polyetheylenimine on polyacrylonitrile UF membranes, while NF membranes with negative charges were produced by grafting sodium 4-styrenesulfonate with halloysite nanotubes on polyethersulfone NF membranes (Zhu et al. 2014; Xu et al. 2016). In these NF membranes, dye removal was consistently over 90% with both positively (624 Da—Bromothymol blue, 452 Da—Orange G, and 408 Da—Crystal violet) and negatively (576 Da—reactive red 49 and 992 Da—reactive black 5) charged membranes (Zhu et al. 2014; Xu et al. 2016).

An alternative to NF membrane is the T-UF membrane, which minimizes the concentration polarization effect of salts and allows better permeability. Indeed, the literature reported that a T-UF membrane of 1700 Da MWCO (made of polydopamine/polyethylenimine with ammonium persulfate on hydrolyzed polyacrylonitrile) could have a dye removal over 98% (reactive orange 1—615 Da, reactive red 2—615 Da, reactive orange 16—618 Da, reactive blue 19—627 Da, reactive blue 4—681 Da, and reactive black 5—992 Da) and a salt permeation over 97% (NaCl and Na_2_SO_4_) (Ye et al. 2020b). Likewise, a T-UF membrane of 4700 MWCO (hydrophilic polyethersulfone) showed a dye rejection over 98.5% (direct red 80—1373 Da, direct red 23—814 Da, congo red—697 Da, and reactive blue 2—840 Da) and salt rejection below 3% (Na_2_SO_4_) (Lin et al. 2016).

Finally, the approach of L-NF membranes is similar to that used in T-UF membranes with a different range of MWCO. These membranes can increase permeability by reducing the concentration polarization effect of salts. In particular, L-NF membranes can be obtained by interfacial polymerization on polyethersulfone UF membranes (Jin et al. 2021, 2022). For example, L-NF membranes (from 780 to 1070 Da) could have a permeability between 52 and 116 L m^−2^ h^−1^ bar^−1^ with a dye rejection over 90% (697 Da—congo red, 814 Da—direct red 23, and 840 Da—reactive blue 2) and salt rejection below 11% (Na_2_SO_4_, NaCl, MgSO_4_, and MgCl_2_) (Jin et al. 2021, 2022).

### Adsorbent nanomaterials

Nanotechnology is receiving great attention in wastewater treatment. The properties and characteristics of nanomaterials, like high aspect ratio, electrostatic properties, tunable pore volume, hydrophobic and hydrophilic properties, reactivity, and high adsorption capacity, make them eligible as very challenging materials in wastewater treatment (Deshpande et al. 2020; Bhat et al. 2022; Chadha et al. 2022). Nanomaterials can effectively eliminate different pollutants like heavy metals, organic and inorganic solvents, colorants, and biological pathogens since nanoparticles may also have antibacterial properties (Madima et al. 2020; Liu et al. 2022).

The main classes of nanocomposite materials comprehend metal oxides, generally employed for heavy metal removal; carbon-based nanomaterials, graphene-based nanoparticles, and nanotubes. Metal and metal oxide are valuable for their high efficiency and low cost. Different studies have shown a suitable metal oxide adsorption of metallic contaminants, such as cadmium, arsenic, chromium, aluminum, etc. (Hu et al. 2005; Boparai et al. 2011; Wen et al. 2014). Magnetic iron oxide nanoparticles, both Fe_3_O_4_ and Fe_2_O_3_ are challenging compounds for various applications thanks to their great chemical stability, safety, and efficiency (Jabbar et al. 2022). Abate et al. synthesized, through a co-precipitation method, humic acid-modified magnetite nanoparticles (HA-Fe_3_O_4_) for cationic malachite-green dye adsorption in wastewater. They evaluated their adsorption efficiency and found that dye adsorption could reach 75 mg g^−1^ HA-Fe_3_O_4_ and 43.69 mg g^−1^ for magnetite nanoparticles without humic acid functionalization. In addition, the synthesized nanoparticles showed good dye-desorption in an HCl water solution, which allowed their regeneration and reuse (Abate et al. 2021).

As far as low-cost adsorbents are concerned, many alternatives can be obtained from industrial-agricultural-domestic wastes/by-products that have been used for dye removal, for example, hair, rice husk, cotton waste, sewage sludge, leaves, coconut shell, fruit peel, clay, marble and living biomass (Gupta and Suhas 2009). In order to reduce secondary pollutions, increase the cycle number of adsorbents and the separation of adsorbents after being used to adsorb dyes, magnetic iron oxide nanoparticles (Fe_2_O_3_ and Fe_3_O_4_) can be incorporated in low-cost adsorbents (Rocher et al. 2008; Zhu et al. 2021). In particular, activated carbon and nanoparticles-Fe_2_O_3_ were dispersed into the polymeric matrix of alginate beads to remove 50% of methylene blue and methyl orange in 10 and 17 min, respectively (Rocher et al. 2008). Likewise, nanoparticles-Fe_3_O_4_ were embedded in marble dust to adsorb 41% of methylene blue from aqueous solution in 90 min at pH 9.0 with a ratio adsorbent-liquid 1200 mg L^−1^ (initial concentration dye 10 ppm) (Ahmed et al. 2022).

Carbon-based nanomaterials are another class of materials having numerous applications in organic and inorganic pollutants adsorption. Carbon nanotubes, for example, possess excellent adsorption thanks to their porosities, hollow and layered structure with internal and external adsorption sites, and the presence of π-conjugated bonds. Moreover, by modifying or functionalizing carbon nanotubes, it is possible to enhance their adsorption properties and selectivity towards a particular class of pollutants. They can interact with the contaminant using electrostatic interaction, ion exchange, surface complexation, and chemical bond formation between the pollutants and the carbon nanotubes (Sajid et al. 2022).

Magnetic carbon nanotubes (M-CNT), prepared by iron oxide deposition onto carbon nanotubes, were employed by Tang et al. to remove microplastics from water. They reported that after 180 min microplastic-magnetic carbon nanotube contact time, the removal efficiency reached 100%. They analyzed polyethylene (PE), polyethylenetereftalate (PET), and polyamide (PA) microplastic adsorption within a suspension containing 5 g L^−1^ M-CNT. The microplastic diameter was about 48 μm. According to this study, different adsorption mechanisms occurred with the three types of microplastics: PE microplastics adsorption was attributed to hydrophobic interactions; PET microplastics were adsorbed on the M-CNT by both hydrophobic as well as π-π interaction, while PA microparticles were adsorbed through complexation, hydrophobic, π-π and electrostatic interactions as well as hydrogen bonding. By a thermal treatment at 600 °C, M-CNT could be recycled and reused (Tang et al. 2021).

Furthermore, carbon nanotubes have been employed mainly in dye removal from wastewater. In a work from Balarak et al., single-walled carbon nanotubes (SWCNTs) were utilized for acid blue 92 dye removal. The authors attributed the adsorption mechanism to hydrogen bonding formation, dipole-induced dipole bonds, besides London, π-π, and hydrophobic interactions. After 75 min pollutant- SWCNTs contact time, with 0.12 g L^−1^ SWCNTs amount, 10 mg L^−1^ dye initial concentration, and pH 3, the dye removal was about 99.4% (Balarak et al. 2021). Mallakpour et al. synthesized hydrogel bio-nanocomposite by tragacanth gum (TG) and carboxyl-functionalized carbon nanotubes through ultrasonication. TG, a biocompatible plant-based polysaccharide, conferred a green character to the process. The synthesized hydrogel bio-nanocomposites were employed for methylene blue dye adsorption. The work showed a 80% dye removal efficiency. Hydrogen bonding, electrostatic, and π-π interactions were the main adsorption mechanisms that occurred (Mallakpour and Tabesh 2021).

Sudhindra et al. combined a polymer-based adsorbent, polyaniline (PANI), with multi-wallet carbon nanotubes (MWCNTs). Polyaniline has received great attention for easy synthesis, environmental stability, and conduction properties. The imine and amine groups in PANI can easily interact with the functional groups of organic dyes, allowing their removal from wastewater. In the study, PANI and MWCNTs were reacted together to prepare a composite through in-situ oxidative polymerization. The azo dye methyl orange was efficiently removed by adsorption on PANI-MWCNTs. The dye removal efficiency reached 98% at 70 °C after 1 h contact time. In addition, the prepared nanocomposites could be reused. They were regenerated after contact with 1 M HCl water solution (Pete et al. 2021). The works reported, and the recent literature have shown adsorbent nanomaterials' great potential in wastewater treatment. However, research is still needed for more in-depth knowledge of nanomaterials, particularly their safety to organisms and the environment.

### Electrolysis

Another technique used in wastewater treatment is electrolysis. This complex method entails the presence of oxidation–reduction reactions for multiple species during the transport of electrical current in the solution. The main advantages of electrolysis are their cost effectiveness and adaptability to different volumes, different types of dyes and a broad range of dye concentration (Raghu and Basha 2007; Radha et al. 2009). In this method, dye degradation is carried out by direct- or indirect oxidation (Vlyssides et al. 2000; Rajkumar and Kim 2006; Wang et al. 2009). The direct-oxidations of dyes are done on the electrode by anodic electron transfer reaction. These reactions may be improved using materials with catalytic activity (graphite, Pt, TiO_2_, Ti/TiO_2_, IrO_2_, PbO_2_, Ti/Pt, Ti/PtO_x_, Ti/Pt–Ir, Ti–Ta–Pt–Ir, Ti/RuO_2_, etc.) (Vlyssides et al. 2000; Chatzisymeon et al. 2006; Rajkumar and Kim 2006; Wang et al. 2009). Meanwhile, the indirect oxidation of dyes takes place when oxidative compounds are produced on the electrode during the electrolysis (Cl_2_, O_2_, ClO_2_, O_3_, H_2_O_2_, and radicals—OH^•^, O^•^, and ClOH^•^) and those diffused into the bulk solution (Vlyssides et al. 2000; Rajkumar and Kim 2006; Wang et al. 2009).

As regards anodic direct-oxidation, it is possible to find Ti/Pt anode that reduced 100% dye content in 18 min (Vlyssides et al. 1999, 2000),Ti-Ta-Pt-Ir with a dye conversion over 80% in 15 min (99% remazol black B, 99% remazol red, 85% remazol golden yellow and 96% cibacron black) (Chatzisymeon et al. 2006), Ti/RuO_2_ with a dye removal of 100% procion black 5B for a flow rate of 10 L h^−1^ (Raghu and Basha 2007),Ti/TiO_2_/RuO_2_/IrO_2_ with a dye removal over 95% at different times for different dyes (21 min—reactive red 120, 28 min—reactive red 141, 8 min——reactive black 5, 12 min—reactive yellow 84, and 6 min—reactive yellow 15) (Rajkumar and Kim 2006) and graphite had a maximum dye removal of 96% (60 min) for an effluent treatment plant at lab-scale (Radha et al. 2009). In the literature, direct cathodic electron transfer is also reported to reduce several textile dyes by a stainless steel multi-cathode and Pt/TiO_2_ anode (Bechtold et al. 2001). Likewise, simultaneous dye removal has been studied by anodic direct/indirect oxidation and indirect cathodic oxidation using graphite as a cathode and Pt/Ti as an anode (Wang et al. 2009). This study showed that after 120 min, the dye removal of a real textile dyeing wastewater is more efficient in the anode than in the cathode, 100% and 35%, respectively (Wang et al. 2009).

In comparison with chemical oxidation methods that employ ozone (dye removal 90%), hypochlorite (dye removal 35%) and Fenton reagent (dye removal > 92%), electrolysis (dye removal 90%) reported after 40 min a good dye degradation using a stainless steel as a cathode and Ti/Pt-Ir as an anode (Szpyrkowicz et al. 2001). In other experiments, it has also been showed that after electrolysis, energy consumption can be reduced by UV light and sunlight (López-Grimau and Gutiérrez 2006). An exposition time of 7 h could increase dye removal compared to darkness, 100% for UV light/sunlight and 90% darkness–electrodes Ti/PtO_X_ and CI reactive orange 4 (López-Grimau and Gutiérrez 2006). In the literature, the integration of solar photovoltaic systems with the electrolysis of textile dyeing wastewater has been studied to obtain hydrogen (Pathak et al. 2020). From this study, it was noticed that the maximum hydrogen production and pollutant removal (between 60 and 96%) was obtained by using steel as an electrode instead of carbon or platinum electrode (Pathak et al. 2020). Finally, it is essential to mention that there are other similar methods to treat wastewater from the textile industry like electrocoagulation (using iron and aluminum electrodes) (Kobya et al. 2003; Bayramoglu et al. 2004) and electrolytic advanced oxidation processes (Nidheesh and Gandhimathi 2014), electro-Fenton (graphite electrodes), peroxicoagulation (cathode graphite and anode iron), and pH-regulated peroxicoagulation (cathode graphite and anode iron) (Nidheesh and Gandhimathi 2014).

## Conclusions

In this bibliometrics analysis, some important aspects of the global research trends in textile wastewater treatment were presented using data from highly cited articles from 1992 to 2021 published in SCIE. The data were related to title, keywords, KeyWords Plus, author keywords, and author performance. From the examination of these data, the main research trends were suggested.

During this time, research into wastewater treatment significantly surged; the annual publication rate almost doubled from 2016 to 2021. Among 386 WoS categories (i.e., toxicology, nanoscience and nanotechnology, fluids and plasmas, composites, and agronomy), multiple researches have tried to develop the best solution for wastewater treatment. Nevertheless, over the past ten years, polymer science journals have published the majority of studies on effluent treatment, showcasing a material-centric approach.

The most common language, English, was used in 97.5% of the total publications, although other languages (14) were also present. Although research on textile wastewater treatment was primarily conducted in one language, it was widely dispersed (97 different countries), with 67 different countries participating in international collaborations, showcasing the global importance of textile wastewater treatment research.

After five countries from Asia, some European nations (Spain, Italy and Poland) were found to be really active in this subject. In the publication of wastewater treatment research, Indian institutions occupied the top spot, followed by Turkish and Chinese universities. The highest CPP_2021_ was recorded by Indian Institute of Technology (47.4).

It has been expected that there would be six key areas of textile wastewater treatment research in the future. In order to find the gold standard for eliminating contaminants in textile effluent, research on textile wastewater treatment is shifting toward the integration of nanomaterials and nanofiltration. The researchers' top choice appears to be adsorbent nanoparticles due to their potential in removing textile colors efficiently from wastewater. The use of improved oxidation methods to remove the colors from textile wastewater is a subject of intense research. Advanced filtration methods based on membranes are currently the subject of extensive research in an effort to make wastewater reusable in the dyeing process.

This bibliometric analysis not only captures the current state of textile wastewater treatment research but also offers a comprehensive perspective on the evolving trends and priorities within the field. As the field continues to evolve, harnessing emerging technologies and fostering global collaboration will be key to securing a cleaner and more sustainable future for the planet earth.

## Data Availability

No data was used for the research described in the article.

## Declarations

### Disclaimer

This manuscript reflects only the authors’ views and opinions, neither the European Union nor the European Commission can be considered responsible for them.
